# Molecular Basis of Macrolide Resistance in* Campylobacter* Strains Isolated from Poultry in South Korea

**DOI:** 10.1155/2018/4526576

**Published:** 2018-07-05

**Authors:** Bai Wei, Min Kang

**Affiliations:** Department of Veterinary Infectious Diseases and Avian Diseases, College of Veterinary Medicine and Center for Poultry Diseases Control, Chonbuk National University, Jeonju, Republic of Korea

## Abstract

We investigated the molecular mechanisms underlying macrolide resistance in 38 strains of* Campylobacter* isolated from poultry. Twenty-seven strains were resistant to azithromycin and erythromycin, five showed intermediate azithromycin resistance and erythromycin susceptibility, and six showed azithromycin resistance and erythromycin susceptibility. Four* Campylobacter jejuni* and six* Campylobacter coli* strains had azithromycin MICs which were 8–16 and 2–8-fold greater than those of erythromycin, respectively. The A2075G mutation in the 23S rRNA gene was detected in 11 resistant strains with MICs ranging from 64 to ≥ 512 *μ*g/mL. Mutations including V137A, V137S, and a six-amino acid insertion (114-VAKKAP-115) in ribosomal protein L22 were detected in the* C. jejuni *strains. Erythromycin ribosome methylase B-*erm*(B) was not detected in any strain. All strains except three showed increased susceptibility to erythromycin with twofold to 256-fold MIC change in the presence of phenylalanine arginine ß-naphthylamide (PAßN); the effects of PAßN on azithromycin MICs were limited in comparison to those on erythromycin MICs, and 13 strains showed no azithromycin MIC change in the presence of PAßN. Differences between azithromycin and erythromycin resistance and macrolide resistance phenotypes and genotypes were observed even in highly resistant strains. Further studies are required to better understand macrolide resistance in* Campylobacter*.

## 1. Introduction

Infection with* Campylobacter* spp. is considered to be the most common cause of bacterial gastroenteritis in humans worldwide. Macrolides are considered the first drug of choice for treating* Campylobacter* gastroenteritis. Resistance to macrolides has been reported in a few scattered clinical isolates of* Campylobacter* across the world. High prevalence of macrolide-resistant* Campylobacter* spp., especially* C. coli,* in animal meat has been reported [1], and this finding is of concern because of the risk of transmission of such isolates to human.

Modification of the antibiotic target genes via methylation or mutation, and efflux of antibiotics from bacterial cells could induce macrolide resistance [2]. The most important macrolide resistance mechanism in* Campylobacter* involves the modification of ribosomal target sites and weakening of the interaction between the tunnel wall of the ribosome and the macrocyclic ring of the macrolide [3]. Point mutations in domain V of the 23S rRNA at positions 2,074 and 2,075 are the most common mechanisms for high-level macrolide resistance in* Campylobacter* spp. [4]. Several modifications in the ribosomal proteins L4 and L22 are associated with low- to intermediate-level macrolide resistance in* Campylobacter* [4]. The chromosomally encoded multidrug resistance-nodulation-cell division (RND) efflux system is involved in intrinsic and acquired macrolide resistance in* Campylobacter* spp. [4]. A ribosomal methylase, encoded by the erythromycin ribosome methylase B-*erm*(B) gene, located in the chromosomal multidrug resistance genomic island (MDRGI) in* C. coli* from swine, was reported for the first time in China in 2014 [5]. Subsequently, several reports emerged of* erm*(B)-harboring* C. jejuni* in animal meat including that of swine and chicken and in human diarrheal samples [6, 7]. Outside China, an* erm*(B)-positive* C. coli* strain was isolated from chicken in Spain in 2016 [8].

Macrolides are a class of natural or semisynthetic products comprising a large macrocyclic lactone ring to which one or more deoxy sugars are attached [2]. The lactone rings can be either 14-membered (clarithromycin, dirithromycin, erythromycin, and roxithromycin), 15-membered (azithromycin), or 16-membered (josamycin, kitasamycin, spiramycin, and tylosin) [2]. In general, modification of ribosomal targets and drug efflux confer cross-resistance to macrolides. During antibiotic treatment in clinical settings, erythromycin and azithromycin are widely used because they have a broad spectrum of activity not only against Gram-positive bacteria, but also against Gram-negative bacteria [9]. Azithromycin is more potent than erythromycin against Gram-negative bacteria including* Campylobacter,* with a lower MIC [9]. However, azithromycin shows a higher MIC than erythromycin against Gram-positive bacteria and rarely acts against Gram-negative bacteria [10, 11]. The diverse mechanisms underlying resistance to erythromycin and azithromycin continue to be unclear.

It is well known that the handling and/or consumption of chicken meat are the main causes of human infection with* Campylobacter*. Other poultry sources also pose a similar threat to human health. Even though the vertical transmission of* Campylobacter* is questionable, breeder chicken harboring antibiotic-resistant bacteria could be a public health threat as they can horizontally transmit antibiotic-resistant* Campylobacter* to broiler chicken in the production chain, indirectly leading to human infection [12]. With the increasing consumption of duck meat around the world and worldwide reports of duck-related product-induced human campylobacteriosis, researchers are focusing more on antibiotic-resistant* Campylobacter* found in duck [13, 14]. Even though the occurrence of highly macrolide-resistant* Campylobacter *in breeder chicken and macrolide-resistant* Campylobacter* in duck meat has been reported [15, 16], the causes of macrolide resistance in these species have rarely been reported. Therefore, we investigated the genetic basis of macrolide resistance in* Campylobacter* from poultry sources including breeder chicken and chicken and duck meat and identified the isolated strains as* C. jejuni* and* C. coli*; these strains showed different levels of resistance to azithromycin and erythromycin, evaluated using molecular methods. Additionally, we sought to investigate the diverse mechanisms underlying the higher resistance to azithromycin, but not to erythromycin, shown by the* Campylobacter* strains.

## 2. Materials and Methods

### 2.1. Origin of* Campylobacter* Strains and Minimal Inhibitory Concentrations (MIC) Determination

A total of 38 strains of* Campylobacter* (15 strains of* C. jejuni* and 23 strains* C. coli*) showing either intermediate resistance or resistance to azithromycin and erythromycin were used in this study (Tables 1 and 2). The strains were isolated from poultry between 2013 and 2016 in a previous study [16]. The sample sources were divided into four types, and the number of strains collected from each source was as follows: (1) in 176 feces samples from breeder chicken farms, 88 isolates of* Campylobacter* were collected and 17 strains (one* C. jejuni* and 16* C. coli*) showed resistance to azithromycin; (2) in 1,003 samples (feces and environmental samples) from broiler chicken farms, 55 isolates were collected and none of them showed resistance to azithromycin or erythromycin; (3) in 249 chicken meat samples from retail markets, 104 isolates were collected and 15 strains (10* C. jejuni* and five* C. coli*) showed resistance to azithromycin; and (4) in 106 duck meat sample from retail markets, 102 isolates were collected and six strains (four* C. jejuni* and two* C. coli*) showed resistance to azithromycin. The MICs of azithromycin and erythromycin were determined using agar or broth dilution methods and the breakpoints were as defined by the National Antimicrobial Resistance Monitoring System (NARMS) for azithromycin: susceptible, ≤ 2 *μ*g/mL; intermediate, 4 *μ*g/mL; and resistant, ≥ 8 *μ*g/mL. The MIC breakpoints for erythromycin were susceptible, ≤ 8 *μ*g/mL; intermediate, 16 *μ*g/mL; and resistant, ≥ 32 *μ*g/mL [17]. The reference strain* C. jejuni* ATCC 33560 was used as the quality control strain.

### 2.2. Characterization of Macrolide Resistance in* Campylobacter *Strains

Genomic DNA templates for PCR were prepared using fresh* Campylobacter* colonies on 5 % sheep blood agar plates (Komed, Seongnam, South Korea) by adding 100 *μ*l sterile distilled water and boiling in a heater block at 100°C for 15 min. Mutations at positions 2,074 and 2,075 of the domain V of 23S rRNA gene were analyzed by sequencing all three copies of 23S rRNA. Three separate reactions were employed to amplify the three copies of the 23S rRNA gene in all* C. jejuni* and* C. coli* strains [3]. Subsequently, potential macrolide resistance-associated mutations were identified by sequencing a 308-bp fragment from each copy of the target gene [3].

In addition, to assess the contribution of mutations within the ribosomal genes* rplD* and* rplV* encoding L4 and L22, respectively, to macrolide resistance, sequence analysis of these genes was performed for all 38 strains. L4- and L22-encoding genes were amplified as previously described [18]. The presence of the recently reported macrolide resistance-related ribosomal RNA methylase gene,* erm*(B), was confirmed using a method described by Zhang et al. [6]. To investigate the role of drug efflux in macrolide resistance,* cmeB, *an efflux pump gene (1,070 bp), was amplified using the method described by Pumbwe et al. [19].

PCR products were purified using JET-SORB gel extraction kit (Genomed, Kampenhout, Belgium) following the manufacturer's instructions. Subsequently, they were sequenced using an ABI 3100 Genetic Analyzer (Applied Biosystems, Foster City, CA). The sequences were analyzed and compared with the reference sequence using the software MEGA (version 5.0). A macrolide-susceptible strain of NCTC 11168 (GenBank: AL111168.1) was used as a reference strain to analyze the mutations in genes encoding 23S rRNA, L4, and L22.

### 2.3. Effects of an Efflux Pump Inhibitor (EPI) on Macrolide Resistance

To investigate the contributions of efflux pump activity to macrolide resistance, the MICs of azithromycin and erythromycin were determined in presence of the EPI phenylalanine arginine ß-naphthylamide (PAßN, Sigma, St. Louis, Missouri). The broth microdilution method was used to determine the MICs in the presence of 20 *μ*g/mL PAßN in Mueller-Hinton broth (Oxoid, Basingstoke, England). The* C. jejuni* ATCC 33560 was used as the reference strain.

## 3. Results

### 3.1. Variation in Resistance to Azithromycin and Erythromycin

Among the 38 tested* Campylobacter* strains, 27 strains, of which nine were* C. jejuni* and 18 were* C. coli,* were resistant to azithromycin and erythromycin (Tables 1 and 2). A total of five strains (three* C. jejuni* and two* C. coli*) showed intermediate resistance to azithromycin with an MIC of 4 *μ*g/mL and were susceptible to erythromycin with MICs ranging from 0.5 to 8 *μ*g/mL. A total of six strains (three* C. jejuni* and three* C. coli*) were resistant to azithromycin and susceptible to erythromycin with MICs ranging from 8–32 *μ*g/mL and 0.5–8 *μ*g/mL, respectively. MICs of azithromycin against four strains of* C. jejuni* (A16-CF-329-3, CM13-MWC-BS-004, A16-CF-127-1-S1, and A16-CF-318-3) and six strains of* C. coli* (A16-CF-319-1, A16-CF-330-1, A16-CF-130-1-S1, DM13-MWC-SS-013, DM13-MP-WS-007, and A16-CF-130-2-S1) were 8–16 and 2–8-fold higher, respectively, than those of erythromycin.

### 3.2. Sequence Analysis of the 23S rRNA

All of the 15* C. jejuni* strains, including one strain (A16-CF-129-1-S1) showing a high-level resistance to erythromycin with an MIC of 128 *μ*g/mL, harbored a wild-type 23S rRNA sequence (Table 1).

The point mutation A2075G was detected in all three copies of the 23S rRNA gene from 11 strains of* C. coli*, which were resistant to azithromycin and erythromycin with MICs ranging from 64 to ≥ 512 *μ*g/mL (Table 2). Two strains of* C. coli *(A16-CF-128-1-S4 and A16-CF-124-2-S3) showed high-level resistance to azithromycin and erythromycin (MIC = 128 *μ*g/mL) and did not harbor any mutations in the 23S rRNA gene, and one strain (A16-CF-128-1-S1) showed high-level resistance to erythromycin (MIC = 128 *μ*g/mL) and did not harbor any mutations in the 23S rRNA gene. The other five strains of* C. coli* were resistant to both azithromycin and erythromycin, with no mutations in the 23S rRNA gene.

### 3.3. Investigation of the Ribosomal Proteins L4 and L22

Analysis of amino acid sequences of the ribosomal proteins L22 and L4 from the* C. jejuni* and* C. coli* strains revealed the presence of different combinations of amino acid substitutions (Tables 1 and 2). The following amino acid substitutions were identified in L4 from* C. jejuni*; V80I, T177S, and M192I in two isolates, V121A in three isolates, and V196A in 12 isolates. The following amino acid substitutions were identified in L22 from* C. jejuni*; I65V in two isolates, A103V in eight isolates, S109A in nine isolates, V137S in one isolate, and V137A in eight isolates. In addition to the amino acid substitutions observed in L22 sequences, a six-amino acid sequence (VAKKAP) present between positions 114 and 115 was also identified in three azithromycin-resistant strains of* C. jejuni*.

Minimal genetic diversity in L4 and L22 amino acid substitutions was observed in 23 strains of* C. coli* from poultry. The substitution V121A was identified in L4 from five* C. coli* strains and the substitution V196A was identified in L4 from 15* C. coli* strains; the substitutions I65V and A74G were identified in L22 from nine* C. coli* strains and the substitutions I65V, A74G, and S109A were identified in L22 from 13* C. coli* strains.

### 3.4. PCR Detection of the* cmeB* and* erm*(B) Gene

The presence of* cmeB* in 14 (93.3 %)* C. jejuni* strains and 4 (17.4 %)* C. coli* strains was confirmed using PCR. However, none of the investigated strains of* C. jejuni* and* C. coli* harbored* erm*(B), as observed in the PCR results obtained.

### 3.5. Efficacy of an EPI

The effects of PAßN on the MICs of macrolide antibiotics in the* C. jejuni* and* C. coli *strains are shown in Tables 1 and 2. The presence of PAßN greatly decreased the MICs of azithromycin and erythromycin against most of* C. jejuni* and* C. coli *strains. In the* Campylobacter* strains with no mutations in the 23S rRNA gene, all the azithromycin intermediate/resistant strains were restored to susceptibility except for four strains (DM13-JDW-WL-009, A16-CF-128-1-S1, A16-CF-130-2-S1, and A16-CF-130-1-S1), and all erythromycin-resistant strains were restored to susceptibility except three strains (DM13-JDW-WL-009, A16-CF-128-1-S1, and A16-CF-130-2-S1).

All the strains showed increased susceptibility to erythromycin in the presence of PAßN with at least two-fold to 256-fold MIC change, except two* C. jejuni* strains and one* C. coli* strain which showed no MIC change. The effect of PAßN on azithromycin MIC was lesser than that on erythromycin MIC, with 13 strains (one* C. jejuni* and 12* C. coli*) of the 38 strains showing no MIC change in the presence of PAßN. In addition, of the 11 strains carrying mutations in the 23S rRNA gene, nine showed no change in azithromycin MIC while all 11 strains showed a 2–16-fold decrease in erythromycin MIC.

## 4. Discussion

In this study,* Campylobacter *strains isolated from breeder chicken and chicken and duck meat between 2013 and 2016 were used to investigate the molecular mechanisms underlying macrolide resistance in* C. jejuni* and* C. coli*. Our data revealed that point mutations at positions 2,075 in domain V of the 23S rRNA gene contributed to high-level azithromycin and erythromycin resistance in 11* Campylobacter* strains. These mutations were not present in* C. jejuni* and* C. coli* strains with a low-level or intermediate resistance to azithromycin and erythromycin. This finding supports previously published reports that suggested a predominant role for this mutation in macrolide resistance [4, 20]. The binding site substitutions of A2075G, A2074G, and A2074C in the 23S rRNA gene in* C. jejuni* and* C. coli* have been implicated in high-level resistance to azithromycin and erythromycin in the field and in the laboratory [4]. The substitution A2075G was the most prevalent genetic mutation conferring high macrolide resistance in the field, suggesting A2075G may provide specific biological or survival advantages compared to A2074G and A2074C [3, 20–22]. Our results were also consistent with those of previous studies in Korea that suggested that the A2075G mutation in the 23S rRNA gene appeared to be the main contributor to high macrolide resistance [23, 24].

As observed in several other species of Gram-negative bacteria, RND efflux pumps confer resistance to macrolides in* Campylobacter* spp. Inhibition of the efflux pumps using EPIs increases the susceptibility of* Campylobacter* to macrolides [20]. Our results showed that an EPI promoted a marked decrease in resistance to both azithromycin and erythromycin in most of* Campylobacter* strains (Tables 1 and 2). In highly erythromycin-resistant strains, the presence of the A2075G 23S rRNA gene mutation and efflux pump activity indicated synergism between these two resistance mechanisms in* Campylobacter* [20]. However, this may not apply to azithromycin because nine out of 11 strains with the A2075G 23S rRNA mutation showed no azithromycin MIC change in the presence of PAßN. Our result was consistent with that of a previous study which found that high azithromycin resistance in* Campylobacter* was mainly due to the A2075G 23S rRNA mutation [25]. These results suggest that the A2075G 23S rRNA mutation in* Campylobacter* was sufficient to confer high-level resistance to azithromycin, while the mutation synergizes with the drug efflux pump system to confer high erythromycin resistance. Further studies are required to assess the contribution of different mechanisms to erythromycin and azithromycin resistance in* Campylobacter*.

A number of previous studies have reported that modifications in the ribosomal proteins L4 and L22 were associated with a lower level of macrolide resistance [26]. Numerous substitutions and insertions in the ribosomal protein sequences in macrolide-resistant strains have been documented. Amino acids at the positions 63–74 are a part of the most important target region in L4; no variation was found in this region of L4 in the present study. In contrast, the most frequent changes, V121A and V196A, were located outside the important target region. The mutations at positions 121 and 196 were identified in susceptible and resistant isolates in previous studies. This suggests that these substitutions are unlikely to contribute directly to macrolide resistance [27]. Other substitutions such as V80I, T177S, and M192I were also identified in erythromycin-susceptible and -resistant isolates previously [23, 27]. In L22, substitutions including I65V, A74G, A103V, and S109A were identified in erythromycin-susceptible and -resistant isolates previously [27, 28]. The mutations V137A and V137S, located in the *β*-hairpin region close to the C-terminus, were identified in* C. jejuni *strains. L22 consists of a small *α* plus *β* domain, with the *β*-hairpin contributing to the formation of the polypeptide tunnel exit at the surface of the ribosome. The mutation in this domain might change the surface properties and block macrolide binding [29]. Nevertheless, isolation of* Campylobacter* strains harboring such substitutions and showing reduced susceptibility to erythromycin did not lead to the elucidation of the mechanistic significance of these amino acid substitutions in the present study.

In addition to single amino acid substitutions, a six-amino acid insertion (114-VAKKAP-115) within the *β*-hairpin region of L22 was found in three* C. jejuni *strains showing azithromycin resistance and reduced susceptibility to erythromycin (Table 1). Amino acid insertions in L22 have been reported in a number of bacterial species, both Gram-negative and Gram-positive [30, 31]. For example, insertions at position 86 or 98 in L22 reportedly conferred macrolide resistance in* C. jejuni *and* C. coli* [26], and a six-amino acid insertion between T108 and V109 in L22 of* Streptococcus* conferred resistance to azithromycin and erythromycin [32]. The significance of this insertion in L22 of* Campylobacter* in relation to macrolide resistance needs to be investigated. In the present study, all strains which showed reduced susceptibility to azithromycin and erythromycin, except one* C. coli* strain, carried the mutations I65V and A74G. These substitutions were not observed in* C. jejuni *strains. The significance of the coexistence of these amino acid substitutions is unknown. The occurrence of such mutations may be associated with local environmental conditions and other selective pressures; most of the previously reported* C. coli* strains, carrying both the amino acid substitutions, were isolated from chicken and swine in Korea [23]. Further studies are required to assess whether the coexistence of these amino acid substitutions contributes to* Campylobacter *macrolide resistance.

Erythromycin, the first 14-membered macrolide, is active against Gram-positive and some Gram-negative microorganisms. To improve acid stability and oral bioavailability of erythromycin, the first 15-membered macrolide, azithromycin, was developed by inserting a basic nitrogen atom into the macrocyclic ring [33]. Azithromycin exhibited enhanced* in vitro* and* in vivo* potency against Gram-positive and Gram-negative bacteria compared to erythromycin [34]. Bacteriostatic and bactericidal activity of azithromycin against* Campylobacter* was up to four times more potent than that of erythromycin [9]. In this study, we found four* C. jejuni* strains and six* C. coli* strains showing higher resistance to azithromycin than to erythromycin; and the MIC of azithromycin was 8–16 and 2–8-fold higher against the* C. jejuni* strains and the* C. coli* strain, respectively, compared to that of erythromycin. Additionally, even in the presence of PAßN, two* C. jejuni* strains (CM13-MWC-BS-004 and A16-CF-318-3) and five* C. coli* (A16-CF-319-1, A16-CF-330-1, A16-CF-130-1-S1, DM13-MWC-SS-013, and A16-CF-130-2-S1) showed higher MICs of azithromycin than of erythromycin. These results were in agreement with those of previous studies in which erythromycin had a lower MIC than azithromycin against both* Campylobacter* and other Gram-positive bacteria [10, 23, 35, 36]. Ribosomal protein polymorphisms might affect the MICs of different macrolides, and an amino acid substitution (A86E) was identified in L22 from azithromycin-resistant and erythromycin-susceptible* Campylobacter* in a previous study [37]. Further, azithromycin was found to be less affected by drug efflux compared with erythromycin, and inactivation of the* cmeB* gene led to greater MIC change of erythromycin than of azithromycin [25]. In the present study, the substitution A86E was not observed in L22 from* C. jejuni* and* C. coli *strains, and higher MICs of azithromycin than of erythromycin were observed even in the presence of an EPI. This may indicate different contributing factors for macrolide resistance, apart from efflux pump-mediated mechanisms. Further studies are required to verify the diverse mechanisms underlying erythromycin and azithromycin resistance and to facilitate the development of strategies to control macrolide-resistant* Campylobacter*. In addition, considering that poultry meat is a common source of human pathogens, careful thought must be given to select effective antibiotics against* Campylobacter* strains from poultry meat showing higher resistance to azithromycin than to erythromycin.

A relatively low prevalence of* cmeB* in* C. coli* strains compared with that in* C. jejuni* strains was found in this study. This was consistent with findings of a recent study on turkeys reported by Olah et al. [38]. The primers used were unable to detect* cmeB* due to the high sequence variation of* cmeB* in* C. coli* isolates [39]. Differences between* C. jejuni* and* C. coli* strains should be further investigated.

The gene* erm*(B) is involved in a major mechanism underlying macrolide resistance in other bacteria, especially Gram-positive bacteria [2]. The transferable* erm*(B) gene located in MDRGI on the chromosome and plasmids with high prevalence in Gram-positive bacteria has been described in highly erythromycin-resistant* Campylobacter* strains [5]. An increase in the incidence of* erm*(B)-carrying* Campylobacter* strains was reported in China, and their prevalence increased from 0.7 % during 2007–2009 to 6.4 % during 2011–2012 [40]. It is noteworthy that a recent study showed a particularly high prevalence (15.1 %) of* erm*(B) in strains in a province of southern China in 2017 [41]. The isolation of an* erm*(B)-carrying clinical* C. jejuni* strain in 1994 suggests that* erm*(B) has a property of diffusional spread along time and space [7]. Following the characterization of* erm*(B)-positive* C. coli* isolated from chicken in Europe, researchers focused on the transferable* erm*(B), located in MDRGI on the chromosome as well as on plasmids. The* erm*(B) gene has not been detected in macrolide-resistant* C. jejuni* and* C. coli* from various sources including human clinical specimens, retail meat, and fecal samples from food animals in the USA or in macrolide-resistant* C. coli* from colon contents of swine in France [37, 42]; in this study,* erm*(B) was not detected in* C. jejuni* and* C. coli* strains from poultry including breeder chicken or chicken and duck meat between 2013 and 2016. Continuous monitoring of* erm*(B) in* Campylobacter* is required, due to its highly transmittable nature.

Recent studies have shown that whole-genome sequencing (WGS) analysis can potentially be a rapid approach to define resistance genotypes and predict resistance phenotypes of bacteria with great sensitivity and specificity, and numerous proof-of-principle studies have also highlighted the value of WGS as a primary diagnostic tool to detect antibiotic resistance [43]. In* Campylobacter*, although a recent study showed that the correlation between resistance phenotypes and genotypes was 100 % in terms of resistance to tetracycline, fluoroquinolones, and erythromycin [37], numerous studies have reported differences between macrolide resistance phenotypes and genotypes of* Campylobacter* from various sources. These studies revealed that* C. jejuni* and* C. coli* strains were resistant to macrolides but did not harbor the corresponding genes or mutations required for resistance [3, 27, 35, 44]. In this study, 27* Campylobacter* strains with both azithromycin and erythromycin resistance did not harbor any mutations within the corresponding genes. Further, three strains were did not harbor any mutation even in the presence of EPI. In such a situation, even after WGS, phenotypic testing would be necessary to confirm macrolide susceptibility of strains given that only deep WGS can detect resistance.

In the present study, we investigated the macrolide resistance of* C. jejuni* and* C. coli* from breeder chicken and chicken and duck meat. Chicken meat is a well-known and a major source for campylobacteriosis in humans; macrolide-resistant* Campylobacter* could be transmitted from duck and breeder chicken to humans. In our study,* Campylobacter* with high resistance to azithromycin and erythromycin was found in breeder chicken. Vertical transmission of* Campylobacter* is questionable and it has been reported that antibiotic-resistant* Campylobacter* from breeder chicken showed clonal homology to that found in humans [1]. In addition, the increased consumption of ducks, especially in Asia, also increases the risk of transmission of antibiotic-resistant* Campylobacter* to humans [13, 14]. Therefore, monitoring populations of macrolide-resistant* Campylobacter* in poultry, including breeder chicken and duck, is required.

It is noteworthy that all* Campylobacter* strains in the present study showing high resistance to azithromycin and erythromycin belonged to a single* C. coli* population isolated from breeder chicken. Similar to a previous study, macrolide resistance has been usually observed in* C. coli*, and it is believed that the high resistance in* Campylobacter* is due to extensive exposure to macrolide derivatives [45]. All* Campylobacter *strains with low or high macrolide resistance were found in breeder chicken from a single integrated company with no difference in management practices or antibiotic usage. This may suggest that polymorphisms, which cause macrolide resistance, could develop even under similar environment pressure. This is in agreement with a previous study in which it was shown that* Campylobacter* utilizes complex and different mechanisms to develop macrolide resistance in the field [46]. Therefore, further studies are required to elucidate mechanisms underlying the development of macrolide resistance in* Campylobacter *during chicken growth.

## 5. Conclusions

In conclusion, the data presented here confirmed previous findings, revealing that a mutation in the 23S rRNA gene at position 2075 showed high-level azithromycin resistance in* Campylobacter* and that the 23S rRNA gene mutation acts synergistically with drug efflux to causes erythromycin resistance. Studies using a larger number of* C. jejuni* and* C. coli* strains showing high resistance to azithromycin but not erythromycin are required to investigate the diverse mechanisms underlying azithromycin and erythromycin resistance in* Campylobacter* spp. Further investigations are required to elucidate the significance of the observed amino acid substitutions V137A and V137S and the six-amino acid insertion identified in L22 from* C. jejuni *strains. None of the investigated strains of* C. jejuni* and* C. coli* from chicken and duck harbored* erm*(B); further surveillance might be required to confirm this. Because differences between macrolide resistance phenotypes and genotypes of* Campylobacter* showing high resistance were found, further studies are needed to improve our understanding of macrolide resistance in* Campylobacter*. To prevent and control macrolide resistance in* Campylobacter*, mechanisms underlying resistance development during chicken growth need to be elucidated. In addition, populations of macrolide-resistant* Campylobacter* in poultry including breeder chicken and duck need to be monitored.

## Figures and Tables

**Table 1:** Characteristics of macrolide resistance genes in *Campylobacter jejuni* strains showing varied resistance to azithromycin and erythromycin^a^.

Strain	Source	MIC (*μ*g/mL)						*cmeB* ^c^	*ermB* ^c^	Mutations			L22 insertions
		Azithromycin			Erythromycin					23S rRNA	L4	L22	
		-EPI	+EPI	Fold^b^	-EPI	+EPI	Fold^b^						
33560	ATCC	0.25	0.06	4	1	0.25	4	nt	nt	wt	wt	wt	wt
A16-CF-329-3	CM	4	0.5	8	0.5	0.5	1	+	-	wt	V196A	A103V, S109A	wt
CM13-HL-010-1	CM	4	0.125	32	8	0.25	32	+	-	wt	V80I, V196A	A103V, S109A, V137A	wt
CM13-HL-012-1	CM	4	0.5	8	8	0.125	64	+	-	wt	V80I, V196A	A103V, S109A, V137A	wt
CM13-MWC-BS-004	CM	8	0.125	64	0.5	0.025	20	+	-	wt	V196A	A103V, S109A, V137A	wt
A16-CF-127-1-S1	BC	8	0.25	32	1	0.25	4	+	-	wt	wt	wt	wt
A16-CF-318-3	CM	8	0.25	32	1	0.015	67	+	-	wt	V121A, T177S, M192I, V196A	V137S	114VAKKAP115
DM13-MP-WS-002	DM	8	0.5	16	64	0.25	256	+	-	wt	V196A	A103V, S109A, V137A	wt
CM13-OP-004-1	CM	8	0.25	32	64	0.5	128	+	-	wt	wt	wt	wt
A16-CF-129-1-S1	CM	8	0.125	64	128	0.5	256	+	-	wt	wt	wt	wt
CM13-HL-BS-021	CM	16	1	16	32	2	16	+	-	wt	V196A	wt	wt
DM13-JDW-WL-007	DM	16	0.25	64	64	0.5	128	-	-	wt	V196A	V137A	114VAKKAP115
DM13-JDW-WL-009	DM	16	16	1	64	64	1	+	-	wt	V121A, T177S, M192I, V196A	S109A, V137A	114VAKKAP115
CM13-MWC-WL-008	CM	16	1	16	64	1	64	+	-	wt	V196A	A103V, S109A, V137A	wt
CM13-DW-BL-005	CM	16	1	16	64	1	64	+	-	wt	V196A	I65V, A103V, S109A, V137A	wt
DM13-JW-SS-017	DM	32	0.5	64	64	0.5	128	+	-	wt	V121A, V196A	I65V, A103V, S109A, V137A	wt

a. Abbreviations: ATCC, American Type Culture Collection; CM, chicken meat; BC, breeder chicken; DM, duck meat; EPI: efflux pump inhibitor; nt: not test; A: Ala; I: Ile; K: Lys; M: Met; P: Pro; S: Ser; V: Val; T: Thr; wt: wild type.

b. Fold values were calculated as MIC without EPI/MIC with EPI.

c. +/-, indicates whether or not the target genes were detected.

The MIC breakpoints for azithromycin: susceptible, ≤ 2 *μ*g/mL; intermediate, 4 *μ*g/mL; and resistant, ≥ 8 *μ*g/mL; for erythromycin: susceptible, ≤ 8 *μ*g/mL; intermediate, 16 *μ*g/mL; and resistant, ≥ 32 *μ*g/mL.

**Table 2:** Characteristics of macrolide-resistance genes in *Campylobacter coli* strains showing varied resistance to azithromycin and erythromycin^a^.

Strain	Source	MIC (*μ*g/mL)						*cmeB* ^c^	*ermB* ^c^	Mutations		
		Azithromycin			Erythromycin					23S rRNA	L4	L22
		-EPI	+EPI	Fold^b^	-EPI	+EPI	Fold^b^					
A16-CF-319-1	CM	4	1	4	1	0.5	2	-	-	wt	wt	wt
A16-CF-330-1	CM	4	1	4	1	0.5	2	-	-	wt	V196A	I65V, A74G
A16-CF-130-1-S1	BC	8	8	1	2	0.5	4	+	-	wt	wt	I65V, A74G
A16-CF-128-1-S1	BC	8	8	1	128	64	2	-	-	wt	V121A, V196A	I65V, A74G
CM13-HL-BS-020	CM	16	0.5	32	32	0.5	64	-	-	wt	V196A	I65V, A74G
CM13-HL-BL-031	CM	16	1	16	64	0.25	256	-	-	wt	V196A	I65V, A74G
CM13-MC-BS-006	CM	16	0.125	128	64	2	32	-	-	wt	V196A	I65V, A74G
DM13-MWC-SS-013	DM	32	2	16	4	0.125	32	-	-	wt	V196A	I65V, A74G
DM13-MP-WS-007	DM	32	0.5	64	8	0.5	16	-	-	wt	V196A	I65V, A74G
A16-CF-130-2-S1	BC	64	64	1	32	32	1	-	-	wt	V196A	I65V, A74G
A16-CF-107-2-S2	BC	64	64	1	64	32	2	-	-	A2075G	wt	I65V, A74G, S109A
A16-CF-128-1-S4	BC	128	2	64	128	4	32	-	-	wt	V196A	I65V, A74G, S109A
A16-CF-124-2-S3	BC	128	4	32	128	2	64	-	-	wt	V196A	I65V, A74G, S109A
A16-CF-107-2-S4	BC	≥512	64	8	≥512	64	8	-	-	A2075G	wt	I65V, A74G, S109A
A16-CF-124-1-S1	BC	≥512	≥512	1	≥512	64	8	-	-	A2075G	wt	I65V, A74G, S109A
A16-CF-124-1-S4	BC	≥512	≥512	1	≥512	64	8	-	-	A2075G	wt	I65V, A74G, S109A
A16-CF-126-2-S1	BC	≥512	64	8	≥512	32	16	-	-	A2075G	wt	I65V, A74G, S109A
A16-CF-124-1-S5	BC	≥512	≥512	1	≥512	64	8	-	-	A2075G	wt	I65V, A74G, S109A
A16-CF-126-2-S2	BC	≥512	≥512	1	≥512	64	8	-	-	A2075G	V196A	I65V, A74G, S109A
A16-CF-126-2-S4	BC	≥512	≥512	1	≥512	64	8	-	-	A2075G	V121A, V196A	I65V, A74G, S109A
A16-CF-118-2-S3	BC	≥512	≥512	1	≥512	64	8	+	-	A2075G	V121A, V196A	I65V, A74G, S109A
A16-CF-126-1-S3	BC	≥512	≥512	1	≥512	32	16	+	-	A2075G	V121A, V196A	I65V, A74G, S109A
A16-CF-126-2-S3	BC	≥512	≥512	1	≥512	32	16	+	-	A2075G	V121A, V196A	I65V, A74G, S109A

a. Abbreviations: CM: chicken meat; BC: breeder chicken; DM: duck meat; EPI: efflux pump inhibitor; A, Ala; G: Gly; I: Ile; S: Ser; V: Val; wt: wild type.

b. Fold values were calculated as (MIC without EPI)/(MIC with EPI).

c. +/-, indicates whether or not the target genes were detected.

The MIC breakpoints for azithromycin: susceptible, ≤ 2 *μ*g/mL; intermediate, 4 *μ*g/mL; and resistant, ≥ 8 *μ*g/mL; for erythromycin: susceptible, ≤ 8 *μ*g/mL; intermediate, 16 *μ*g/mL; and resistant, ≥ 32 *μ*g/mL.

## Data Availability

Sequence data that support the findings of this study have been deposited in the GenBank with the accession number MH084527-MH084640.
